# Effective treatment of advanced Oropouche virus, Rift Valley fever virus, and Dabie bandavirus infections with 4'-fluorouridine

**DOI:** 10.1128/mbio.01467-25

**Published:** 2025-09-12

**Authors:** Jonna B. Westover, Kie Hoon Jung, Inioska Rojas, Kevin W. Bailey, Julio Landinez-Aponte, Gregory R. Blumeling, Shuli Mao, Alexander A. Kolykhalov, Michael G. Natchus, George R. Painter, Brian B. Gowen

**Affiliations:** 1 Department of Animal, Dairy and Veterinary Sciences, Institute for Antiviral Research, Utah State Universitys 4606, Logan, Utah, USA; 2 Emory Institute for Drug Development, Emory University, Atlanta, Georgia, USA; Virginia Tech, Blacksburg, Virginia, USA

**Keywords:** antiviral agents, human pathogen, bunyavirus, rodent model, public health

## Abstract

Oropouche virus (OROV), Rift Valley fever virus (RVFV), and Dabie bandavirus (DBV) are significant re-emerging and emerging human pathogens with major public health implications. Notably, the ongoing OROV disease epidemic spanning South America, Central America, and the Caribbean now exceeds 11,000 cases, including several fatalities and reports of neurological disease and congenital abnormalities associated with infection. Rift Valley fever outbreaks continue to plague sub-Saharan Africa, and DBV, the etiologic agent of severe fever with thrombocytopenia syndrome (SFTS), is expanding its reach throughout several Asian countries. No vaccines or approved therapies are available to prevent or treat these viral infections. Here, we report on the antiviral activity and protective efficacy of the ribonucleoside analog, 4′-fluorouridine (4′-FlU), against OROV, RVFV, and DBV in cell culture and murine models of infection and disease. In cell culture, the potency of 4′-FlU was in the low nanomolar (OROV) to low micromolar (RVFV and DBV) range. In vivo, prophylactic oral dosing of the compound was fully protective against all three viruses in their respective mouse infection models. Importantly, post-exposure and therapeutic interventions of advanced infections in mice also responded remarkably well to treatments. Our findings extend the broad-spectrum antiviral capacity of 4′-FlU and support the compound’s further development for treating severe bunyaviral infections.

**IMPORTANCE:**

Re-emerging and emerging viral diseases, for which no approved vaccines or therapeutics exist, pose a significant public health threat in affected areas of the world. Antiviral drugs that are broadly active against multiple pathogenic viruses are much needed. Our findings demonstrating robust protection conferred by treatment with 4′-fluorouridine (4′-FlU) in viral infection models for Oropouche fever, Rift Valley fever, and severe fever with thrombocytopenia syndrome support the continuing development of this promising broad-spectrum antiviral drug candidate for the treatment of these notable viral diseases.

## INTRODUCTION

Oropouche virus (OROV; *Peribunyaviradae, Orthobunyavirus*), Rift Valley fever virus (RVFV; *Phenuiviridae, Phlebovirus*), and Dabie bandavirus (DBV; *Phenuiviridae, Bandavirus*) are arthropod vector-transmitted bunyaviruses that pose significant public health threats in regions of the world where they are endemic. Infection by these viruses can lead to severe febrile disease and, in some cases, death. The current OROV disease outbreak has reached unprecedented levels in South and Central America and the Caribbean, with the first deaths and cases describing vertical transmission, neurological disease, and fetal complications reported recently (1–4). OROV is transmitted primarily through biting midges and, to a lesser extent, mosquitoes (2). The rising incidence of cases prompted the Pan American Health Organization (PAHO) and World Health Organization (WHO) to issue multiple epidemiological alerts in 2024 (5). By September, there were nearly 10,000 confirmed OROV infection cases in 11 countries, including Bolivia, Brazil, Colombia, Cuba, Dominican Republic, and Peru, with imported cases in Canada, Germany, Italy, Spain, and the USA (6, 7). First reported in 1955 in Trinidad and Tobago (8), outbreaks of OROV disease have now spread to areas with no previous history (7), a warning that it is likely to continue expanding its geographical range.

RVFV causes a severe hemorrhagic fever (colloquially known as Rift Valley fever; RVF) that can present with a devastating neurologic component and, in some cases, infection in the eyes can lead to varying severity of vision loss (9, 10). RVF was first described in 1931 in the Rift Valley of Kenya, with many outbreaks of disease in humans and vulnerable livestock species occurring since then (11). Originally confined to sub-Saharan Africa, the geographical footprint of RVFV has expanded with outbreaks of disease in humans and livestock reported in the Arabian Peninsula and off the coast of East Africa in Comoros, Mayotte, and Madagascar. The virus is spread by various species of mosquitoes or contact with infected animal tissues (12, 13). RVF is a WHO R&D Blueprint priority disease (14), highlighting global concerns over the spread of the virus, with the potential to impact human health and agriculturally important livestock species. Another WHO pathogen of concern is DBV, commonly known as severe fever with thrombocytopenia syndrome (SFTS) virus (14, 15). The virus has emerged in several Asian countries, causing life-threatening disease with case fatality rates approaching 30% in hospitalized individuals (16). First described in 2009 in China (17), cases of SFTS have now been reported in eastern and southeast Asia, including South Korea, Japan, Taiwan, Thailand, Myanmar, and Vietnam (18, 19). With the widening distribution of the primary tick vector, *Haemaphysalis longicornis*, including its recent introduction into the USA, there are growing concerns about further expansion of DBV into new areas of the world (20–22). Unfortunately, no FDA-approved antiviral drugs or vaccines are available to treat or prevent these medically important emerging and re-emerging vector-borne, bunyaviral diseases.

OROV, RVFV, and DBV have tri-segmented, single-stranded negative-sense RNA genomes encoding a limited arsenal of proteins. The small (S) genomic segments of these viruses encode N protein and a nonstructural protein (NSs) (23). For OROV, the NSs is translated from within the N gene mRNA transcript (7), whereas RVFV and DBV employ an ambisense coding strategy transcribing the NSs viral mRNA from the antigenomic strand (23). Generally, the N proteins encapsulate the viral genome segments and are required for viral nucleic acid transcription and replication, whereas the NSs proteins antagonize the host innate immune response (7, 23–25). The medium (M) genomic segment of these viruses encodes the viral glycoprotein complex consisting of Gn and Gc proteins, which facilitate cellular attachment and entry (23). In addition to the Gn/Gc complex, the M segments of RVFV and DBV encode a nonstructural protein, NSm, and, in the case of RVFV, a 78-kD protein (23, 25). Encoded on the large (L) genomic segments is the multifunctional L protein, which has both RNA-dependent RNA polymerase (RdRp) and endonuclease activity (23, 26). Central to viral mRNA transcription and genome replication is the RdRp activity. In addition to the lack of a host homolog and structural conservation, this function makes the RdRp an attractive antiviral drug target with a high genetic barrier to resistance.

The ribonucleoside analog, 4′-fluorouridine (4-FlU; also known as EIDD-2749), is structurally similar to uridine but with a fluorine atom at the 4′ position. The active triphosphate form (4′-FlU-TP) has been shown to be an immediate chain terminator of influenza A virus (IAV) RdRp (27), while causing transcriptional stalling of respiratory syncytial virus (RSV) and SARS-CoV-2 RdRps (28). Additionally, 4′-FlU has shown promise as a broad-spectrum antiviral drug with reported efficacy in rodent models of respiratory viral infections (RSV, IAV, and SARS-CoV-2) (27, 28), arenaviral hemorrhagic fevers (Lassa and Junín viruses) (29), and several arboviral diseases (Heartland and Chikungunya viruses) (30, 31). Here, we report on the antiviral activity of 4′-FlU against OROV, RVFV, and DBV in cell culture and mouse models of infection and disease. In cell culture-based assays, OROV was exquisitely sensitive to 4′-FlU at a low nanomolar concentration, whereas the compound was active in the low micromolar range against RVFV and DBV. *In vivo*, prophylactic intervention with 4′-FlU prevented disease development at remarkably low doses, and the compound maintained its efficacy with post-exposure and therapeutic application, effectively reversing disease when starting treatment after the onset of weight loss in the SFTS disease model.

## RESULTS

### *In vitro* activity of 4′-FlU vs. RVFV, DBV, and OROV

The antiviral activity of 4′-FlU was first evaluated in cell-based RVFV, DBV, and OROV infection models by virus yield reduction (VYR) assays. Cells were treated with escalating serial log_10_ dilutions of 4′-FlU just prior to virus infection. Ribavirin and favipiravir were included as positive controls. The 90 percent inhibitory concentration (EC_90_) values, expressed as the drug concentration that reduced virus yield by one log_10_, of 4′-FlU against RVFV and DBV were in the low-micromolar to high-nanomolar range, 18-fold to 70-fold and 16-fold to 29-fold more potent than ribavirin and favipiravir, respectively (Table 1). Against OROV, the compound’s potency was in the low-nanomolar range (7.4 nM), >8,000 times more potent than ribavirin and 2297 times more potent than favipiravir. The selectivity index (SI_90_) for all three viruses exceeded 90.

**TABLE 1:** *In vitro* inhibition of RVFV, DBV, and OROV by 4′-FlU and other viral polymerase-targeting compounds^*a*^^,^^*b*^

		4′-FlU	Ribavirin	Favipiravir
RVFV	EC_90_ (μM)	3.5 ± 0.39	64 ± 16	55 ± 1.8
	CC_50_ (μM)	330 ± 35	3863 ± 401	> 3183
	SI_90_	95 ± 3.5	62 ± 15	> 175 ± 17
DBV	EC_90_ (μM)	0.82 ± 0.10	57 ± 8.4	24 ± 2.2
	CC_50_ (μM)	> 381	> 4095	> 3183
	SI_90_	> 471 ± 51	> 72.8 ± 11	> 133 ± 12
OROV^*c*^	EC_90_ (μM)	0.0074 ± 0.0017	60 ± 13	17 ± 1.0
	CC_50_ (μM)	> 1	3767 ± 321	3122 ± 106
	SI_90_	> 141 ± 36	64 ± 8.2	188 ± 7.2

^*a*^ The inhibitory effect of 4′-FlU, ribavirin, and favipiravir was evaluated in 4-day VYR assays using Vero 76 (RVFV) or Vero E6 (DBV, OROV) cells. The data represent the mean ± standard deviation of 3 separate experiments.

^*b*^ EC_90_ = 90% inhibitory concentration. CC_50_ = 50% cell cytotoxic concentration. SI_90_, Selectivity Index = CC_50_/EC_90_.

^*c*^ Strain BeAn 19991.

### Prophylactic efficacy of oral 4′-FlU in bunyavirus mouse infection models

**Fig 1:**
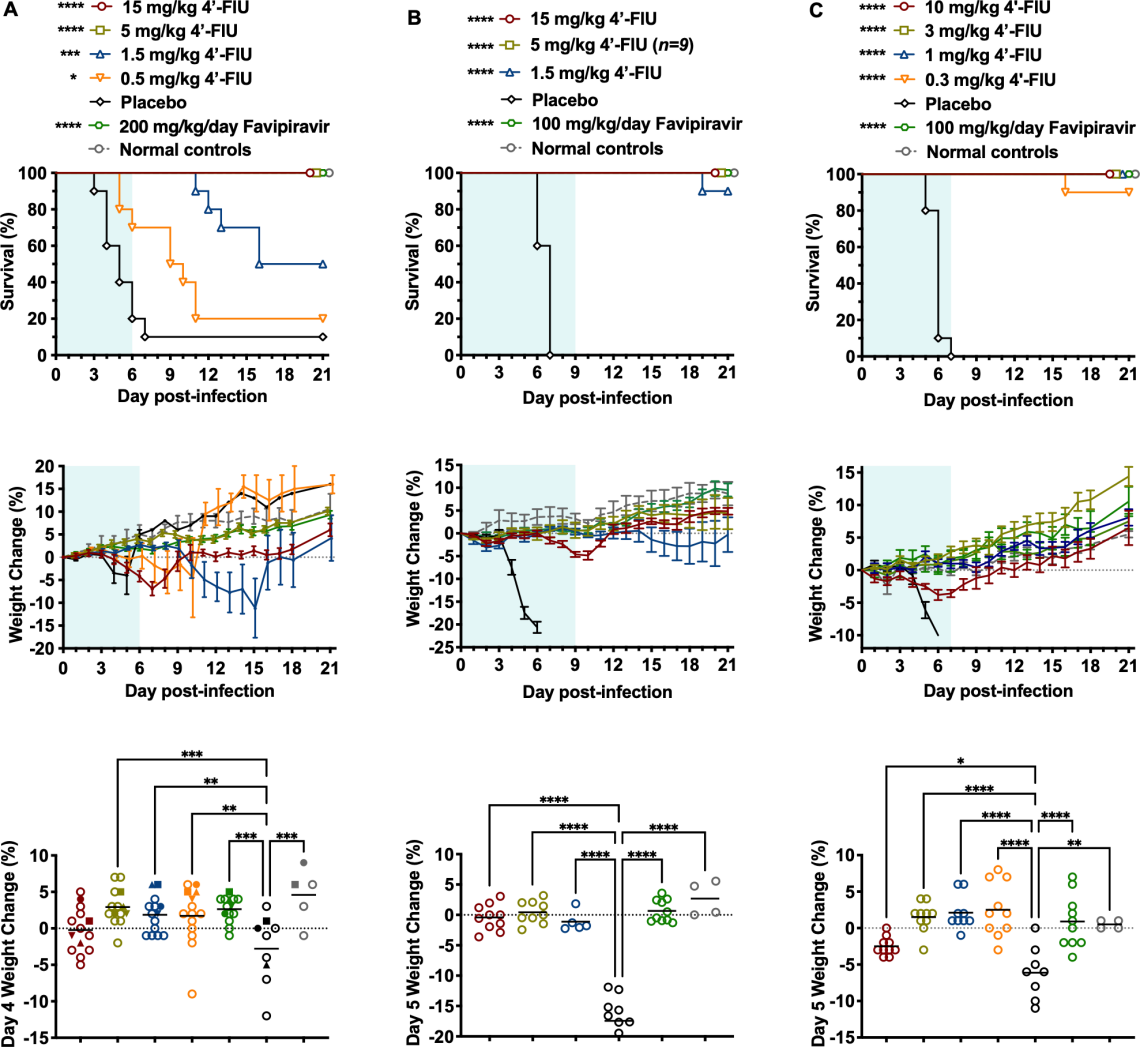
Effect of 4′-FlU on survival outcome and body weights of mice treated prophylactically and challenged with RVFV, DBV, or OROV. Animals in each group were treated p.o., QD with the indicated dose of 4′-FlU, placebo, or positive control drug (*n* = 10, unless otherwise indicated) initiated within 2 h of each infection. (**A**) BALB/c mice challenged with RVFV, (**B**) *Ifnar^-/-^* mice challenged with DBV, and (**C**) *Ifnar^-/-^* mice challenged with OROV. The weight data are represented as the group mean and standard error of the percent change in weight of surviving animals relative to their starting weights on the day of virus challenge. Solid symbols in the single-day RVFV weight loss graph represent the same animals in Fig. 2A. Sham-infected (MEM only) normal control animals (*n* = 4) are shown for comparison. Blue-shaded areas denote the treatment durations. ****P < 0.0001, ***P < 0.001, **P < 0.01, *P < 0.05 compared with animals that received the placebo.

Having demonstrated low-nanomolar to low-micromolar antiviral activity against pathogenic RVFV, DBV, and OROV in cell culture (Table 1 and Fig. S1) and favorable oral bioavailability in mice (27), the ability of 4′-FlU to protect against the highly lethal viral infections in their respective mouse models was evaluated. For the RVFV BALB/c infection model, the 4′-FlU compound was administered by oral gavage (p.o.), once daily (QD) for 7 days, beginning 2 h prior to subcutaneous (s.c.) challenge. As shown in the top panel of Fig. 1A, all animals treated with 15 or 5 mg/kg doses of 4′-FlU survived the RVFV infection that resulted in 90% mortality in the placebo mice. Efficacy waned in a dose-responsive manner in animals treated with 1.5 and 0.5 mg/kg of the compound, providing 50% and 20% protection, respectively, with extended survival time in many of the mice that succumbed to the RVFV infection. The high-dose favipiravir treatment provided complete protection against the lethal RVFV challenge, and the mice had a longitudinal weight gain trajectory similar to that of the group of animals treated with 5 mg/kg 4′-FlU (Fig. 1A, middle panel). Notably, the group of mice that received the 15 mg/kg dose of 4′-FlU began losing weight on day 4, with recovery starting shortly after the final treatments were completed on day 6 post-infection (p.i.), suggesting tolerability issues at this highest tested dose. The two groups of mice administered the lower doses of 4′-FlU experienced weight loss consistent with the survival data. To assess weight loss at a time during the acute infection when most placebo-treated animals could be included in the analysis, the day-4 percent weight change of all mice in the study, including those that were analyzed for serum and tissue viral titers, is shown in the bottom panel of Fig. 1A. Groups of mice in 0.5 to 5 mg/kg 4′-FlU dose range, but not the 15 mg/kg group, had significantly improved weight profiles on day 4 p.i. compared with the placebo group.

The prophylactic efficacy of escalating oral doses of 4′-FlU was also evaluated in the DBV *Ifnar^-/-^* mouse infection model, extending the treatment period to 10 days based on the slower-evolving disease compared with RVFV infection. As observed in the RVFV mouse infection model, oral dosing of 15 and 5 mg/kg of 4′-FlU was fully protective (Fig. 1B, top panel). The lowest tested dose of 1.5 mg/kg was also highly efficacious, protecting 9 of 10 mice from a uniformly lethal challenge dose and dramatically altering the disease course in the mouse that succumbed on day 19 p.i. As expected, the high-dose favipiravir positive control treatment provided complete protection against the lethal s.c. DBV challenge. The severity of DBV infection in *Ifnar^-/-^* mice treated with the vehicle placebo is underscored by the precipitous weight loss beginning 4 days after challenge through the time when they succumb 6–7 days p.i. (Fig. 1B, middle panel). Mice treated with 5 mg/kg 4′-FlU or favipiravir had similar weight gain trajectories, with the animals in the 15 mg/kg 4′-FlU group experiencing modest weight loss during the second week of the infection before bouncing back within a few days of the final treatment. The reduced mean weight of the 1.5 mg/kg 4′-FlU group at the latter stage of the study resulted from several mice with mild-to-moderate weight loss, including the mouse that succumbed on day 19 p.i. All surviving animals appeared healthy and began gaining weight when the study concluded on day 21. The significant protective effect conferred by the 4′-FlU treatments against acute disease and weight loss in DBV-infected mice was most prominent on day 5 p.i. (Fig. 1B, bottom panel). The reversible weight loss observed in the initial experiments in mice treated with 15 mg/kg indicates a potential tolerability issue. To limit complications that might arise from toxicity, doses were kept at 10 mg/kg or lower in the following experiments.

In the lethal *Ifnar^-/-^* mouse BeAn 19991 strain OROV infection model, oral 4′-FlU was also highly efficacious with early intervention. Because of the greater sensitivity of OROV to 4′-FlU (Table 1), lower doses ranging from 0.3 to 10 mg/kg and a 7-day treatment regimen were investigated. As shown in the top panel of Fig. 1C, except for one animal in the lowest 0.3 mg/kg dose group, all the 4′-FlU-treated mice survived the OROV challenge, whereas animals treated with the placebo succumbed within 7 days p.i. The severity of the acute disease and the significant protective effect from the 4′-FlU treatment are highlighted by the sharp decline in body weight in the placebo-treated animals on day 5 p.i. (Fig. 1C, middle and bottom panels). In contrast, body weights were stable or increased in animals treated with 0.3, 1, or 3 mg/kg of 4′-FlU or the favipiravir positive control drug. Furthermore, despite lowering the previously tested highest dose of 4′-FlU from 15 mg/kg (RVFV, DBV) to 10 mg/kg for the OROV study, a temporary and shallow decline in body weight was again observed in the mice receiving the highest dose of the compound, with the mice recovering from the weight loss following the cessation of treatment.

**Fig 2:**
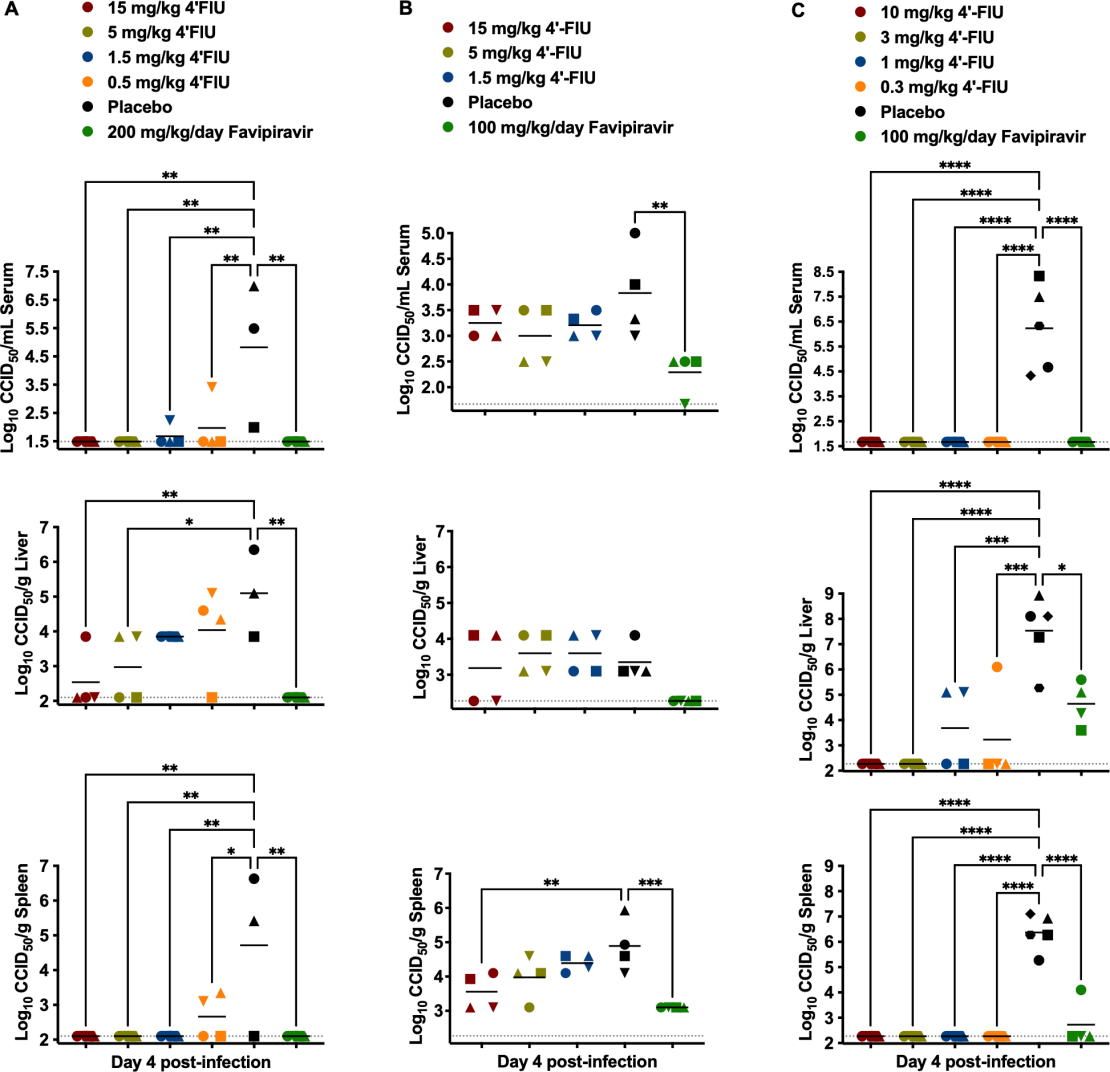
Analysis of day 4 viremia and tissue viral titers in virus-infected mice treated with 4′-FlU. Mice were treated prophylactically with 4′-FlU then challenged with (**A**) RVFV, (**B**) DBV, or (**C**) OROV. Subsets of animals in each group (*n* = 4-5) were designated for sacrifice on day 4 p.i. to assess infectious virus titers in the serum, liver, and spleen tissues. Unique symbols in each treatment group represent values for the same animal across all parameters. Horizontal lines represent the mean for each group. The dotted lines represent the assays’ lower limits of detection. ****P < 0.0001, ***P < 0.001, **P < 0.01, *P < 0.05 compared with animals that received the placebo.

The effect of 4′-FlU treatments on limiting viremia and liver and spleen tissue viral titers in mice challenged with RVFV, DBV, or OROV was also determined in subsets of mice sacrificed on day 4 p.i. during the prophylactic efficacy studies. As shown in Fig. 2A, all tested doses of 4′-FlU resulted in a significant reduction in serum and spleen RVFV loads, and the 5 and 15 mg/kg doses also significantly reduced the viral burden in the liver samples. Against DBV, significantly reduced viral titers were only observed in the spleen samples at the 15 mg/kg dose of 4′-FlU, whereas favipiravir significantly reduced serum and spleen viral loads (Fig. 2B). As shown in Fig. 2C, OROV-challenged mice had notably higher viral titers than DBV on day 4 p.i. In stark contrast to mice treated with the placebo, all 4′-FlU treatments resulted in undetectable infectious virus in serum and spleen samples and significant reductions in viral loads in the liver. Favipiravir was also effective, reducing viral burden in the serum, spleen, and, to a lesser extent, the liver.

### Post-exposure and therapeutic efficacy of 4′-FlU in treating advanced bunyavirus infections in mice

**Fig 3:**
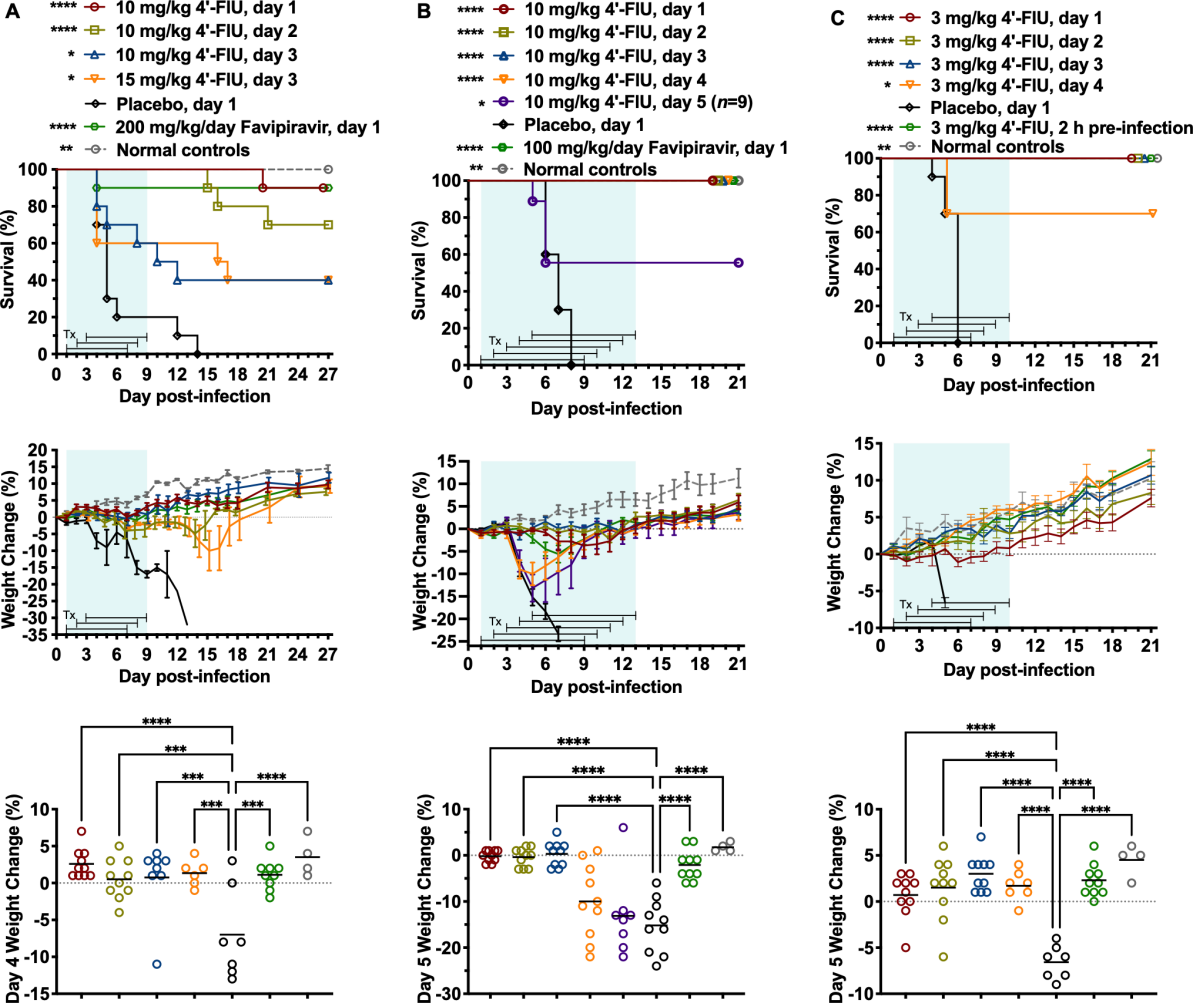
Effect of delayed 4′-FlU treatment on survival outcome and daily weights of mice challenged with RVFV, DBV, or OROV. Animals in each group (*n* = 10, unless otherwise indicated) were treated p.o., QD with the indicated dose of 4′-FlU, placebo, or positive control drug. (**A**) BALB/c mice challenged with RVFV. (**B**) *Ifnar^-/-^* mice challenged with DBV. (**C**) *Ifnar^-/-^* mice challenged with OROV. The weight data are represented as the group mean and standard error of the percent change in weight of surviving animals relative to their starting weights on the day of virus challenge. Sham-infected (MEM only) normal control animals are shown for comparison. Blue-shaded areas define the treatment (Tx) range, and brackets indicate specific group Tx durations. ****P < 0.0001, ***P < 0.001, **P < 0.01, *P < 0.05 compared with animals that received the placebo.

Having observed remarkable protection against RVFV, DBV, and OROV infections with prophylactic dosing of 4′-FlU, the time window for effective intervention was explored by delaying the time of treatment initiation in each of the disease models. For RVFV infection, a dose of 10 mg/kg 4′-FlU, beginning 1, 2, or 3 days p.i., was selected to adjust for the weight loss observed in the prophylactic efficacy study at the 15 mg/kg 4′-FlU dose during the latter part of the treatment course. However, the 15 mg/kg dosing regimen was also explored with the longer 3-day delay in treatment initiation. When treatments began 1 day p.i., 90% protection was afforded (Fig. 3A, top panel). When treatments were delayed until 2 days p.i., 70% protection was observed. In the day 1 and 2 treatment initiation groups, the mice that succumbed survived 9–22 days longer than the majority (80%) of the placebo-treated mice, which all succumbed by day 14 p.i. Further delaying 4′-FlU treatment until day 3 p.i., when many mice were sick and showing signs of disease, 4′-FlU still provided statistically significant protection (*P* < 0.05), with 40% survival observed in groups treated with 10 or 15 mg/kg of the compound. The longitudinal weight data are generally consistent with the survival outcome (Fig. 3A, middle panel), and specifically looking at the collective body weights on day 4 p.i., significant protection is observed with all 4′-FlU and favipiravir groups (Fig. 3A, lower panel).

With DBV infection in *Ifnar^-/-^* mice, 10 mg/kg treatment regimens of 4′-FlU were started at day 1, 2, 3, 4, or 5 p.i., with the latter start times coinciding with the onset of clinical disease. Animals receiving 4′-FlU beginning on days 1–4 after virus challenge survived the lethal DBV challenge (Fig. 3B, top panel). Remarkably, significant therapeutic protection (56% survival; *P* < 0.05) was observed in the mice treated with 4′-FlU starting 5 days p.i., one day after the onset of weight loss (Fig. 3B, middle panel). In contrast, uniform lethality and unchecked precipitous weight loss were seen in mice treated with the vehicle placebo. As expected, the positive control favipiravir treatment initiated on day 1 p.i. provided complete protection against DBV challenge with modest weight loss from days 5-7 before rebounding starting on day 8 p.i. Notably, the mice that received 4′-FlU beginning on days 4 or 5 p.i. lost a substantial amount of weight until they began treatment, rapidly reversing the weight loss. All surviving animals were in good health with upward weight gain trajectories at the conclusion of the study. The efficacy of the post-exposure treatments was also exemplified by the dramatic difference in day 5 body weights between mice treated with 4′-FlU starting 1–3 days p.i., compared with those treated with the vehicle placebo (Fig. 3B, bottom panel).

For the OROV BeAn 19991 strain disease model, post-exposure intervention with a lower dose of 3 mg/kg of 4′-FlU was evaluated starting 1, 2, 3, or 4 days after virus challenge. Treatment with the same dose initiated 2 h p.i. was included as the positive control and provided 100% protection of *Ifnar^-/-^* mice against the uniformly lethal challenge dose of OROV (Fig. 3C, top panel). Starting treatments 1–3 days after OROV challenge afforded complete protection against mortality and prevented the sharp decline in body weight observed in the placebo-treated mice on day 5 p.i. (Fig. 3C, middle panel). All animals receiving the vehicle placebo succumbed to the disease within 6 days of the virus challenge. Impressively, 70% of animals that started treatment with 3 mg/kg 4′-FlU at or near the onset of illness on day 4 p.i. survived and fully recovered from the otherwise lethal OROV challenge. The protective antiviral effect was also documented in the comparison of day-5 body weights, with the 4′-FlU mitigating weight loss (Fig. 3C, bottom panel).

**Fig 4:**
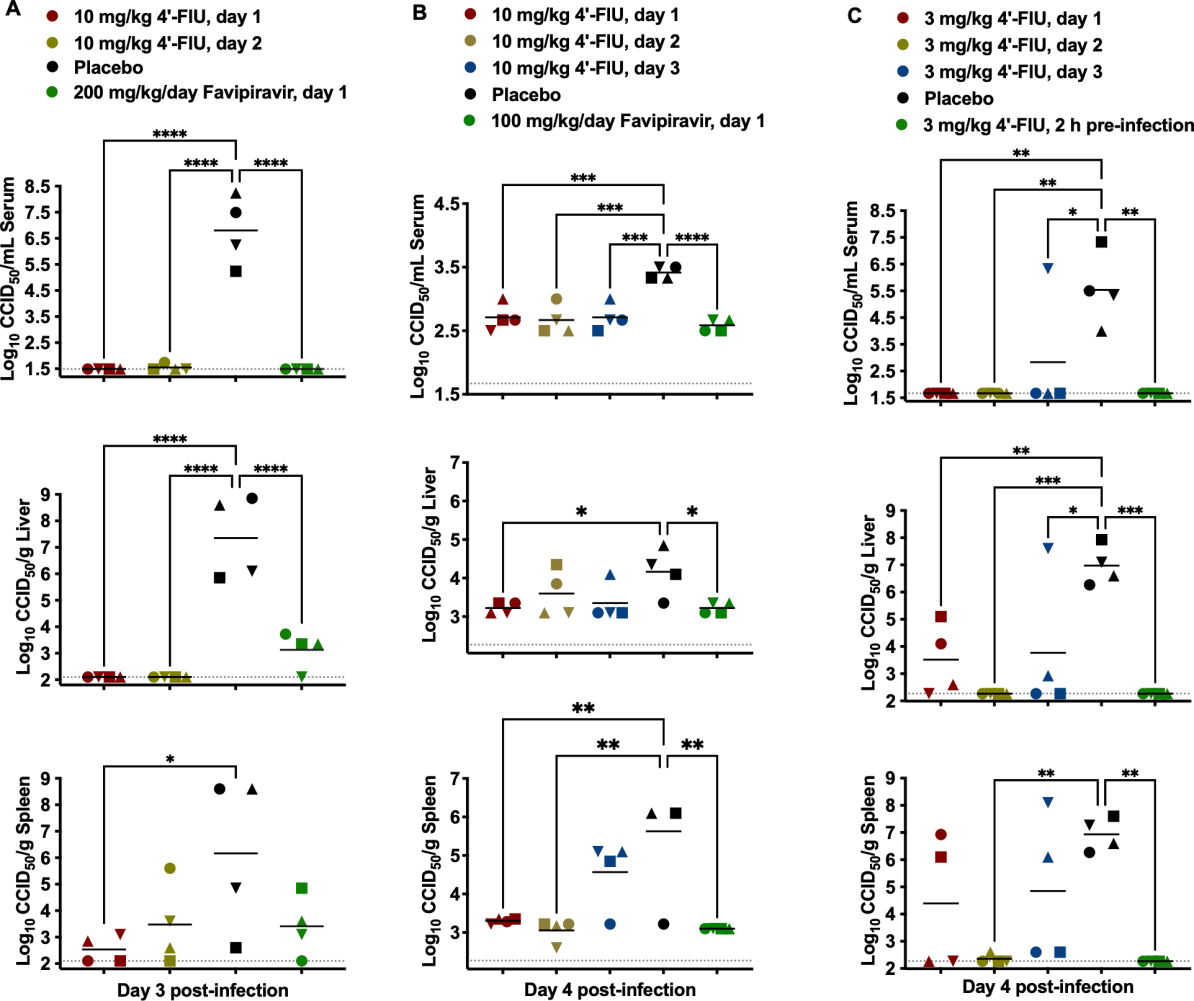
Analysis of viremia and tissue viral titers in virus-challenged mice treated post-exposure with 4′-FlU. Mice were challenged with (**A**) RVFV, (**B**) DBV, or (**C**) OROV, then treated with 4′-FlU beginning on the indicated day p.i. Subsets of animals in each group (*n* = 4) were designated for sacrifice on day 3 or 4 p.i. to assess infectious virus titers in the serum, liver, and spleen tissues. Unique symbols in each treatment group represent values for the same animal across all parameters. Horizontal lines represent the mean for each group. The dotted lines represent the assays’ lower limits of detection. ****P < 0.0001, ***P < 0.001, **P < 0.01, *P < 0.05 compared with animals that received the placebo.

As with the prophylactic efficacy studies, the impacts of 4′-FlU treatments on RVFV, DBV, OROV serum, liver, and spleen viral titers were assessed. RVFV viremia was mostly undetectable on day 3 p.i. in mice treated with 10 mg/kg 4′-FlU or 200 mg/kg/day favipiravir (Fig. 4A, top panel). RVFV titers in liver tissue were only detectable in the placebo-treated mice and, to a significantly lesser extent, in the favipiravir-treated animals (Fig. 4A, middle panel). The spleen samples, however, were less distinct, with infectious virus detectable in all experimental groups and the placebo-treated mice having the highest viral loads (Fig. 4A, bottom panel). In the case of DBV infection, the 10 mg/kg 4′-FlU treatment significantly (*P* < 0.001) limited the low-level viremia observed in the placebo-treated mice, similar to the reduction by high-dose favipiravir (Fig. 4B, top panel). A significant effect was also observed with the 10 mg/kg 4′-FlU dose in the liver and spleen tissues. With OROV, the 3 mg/kg 4′-FlU treatments were mostly effective at reducing day 4 viremia and tissue viral loads, except for a single animal (inverted blue triangle) in the day-3 treatment initiation group, which had high viral loads in all samples comparable to the placebo-treated mice (Fig. 4C). Overall, viral titers (or lack thereof) in mice treated with 4′-FlU were comparable or superior to favipiravir in the blood and selected tissues and, in most cases, significantly reduced viral burdens.

### Number of 4′-FlU doses required to achieve therapeutic efficacy in the DBV infection model

**Fig 5:**
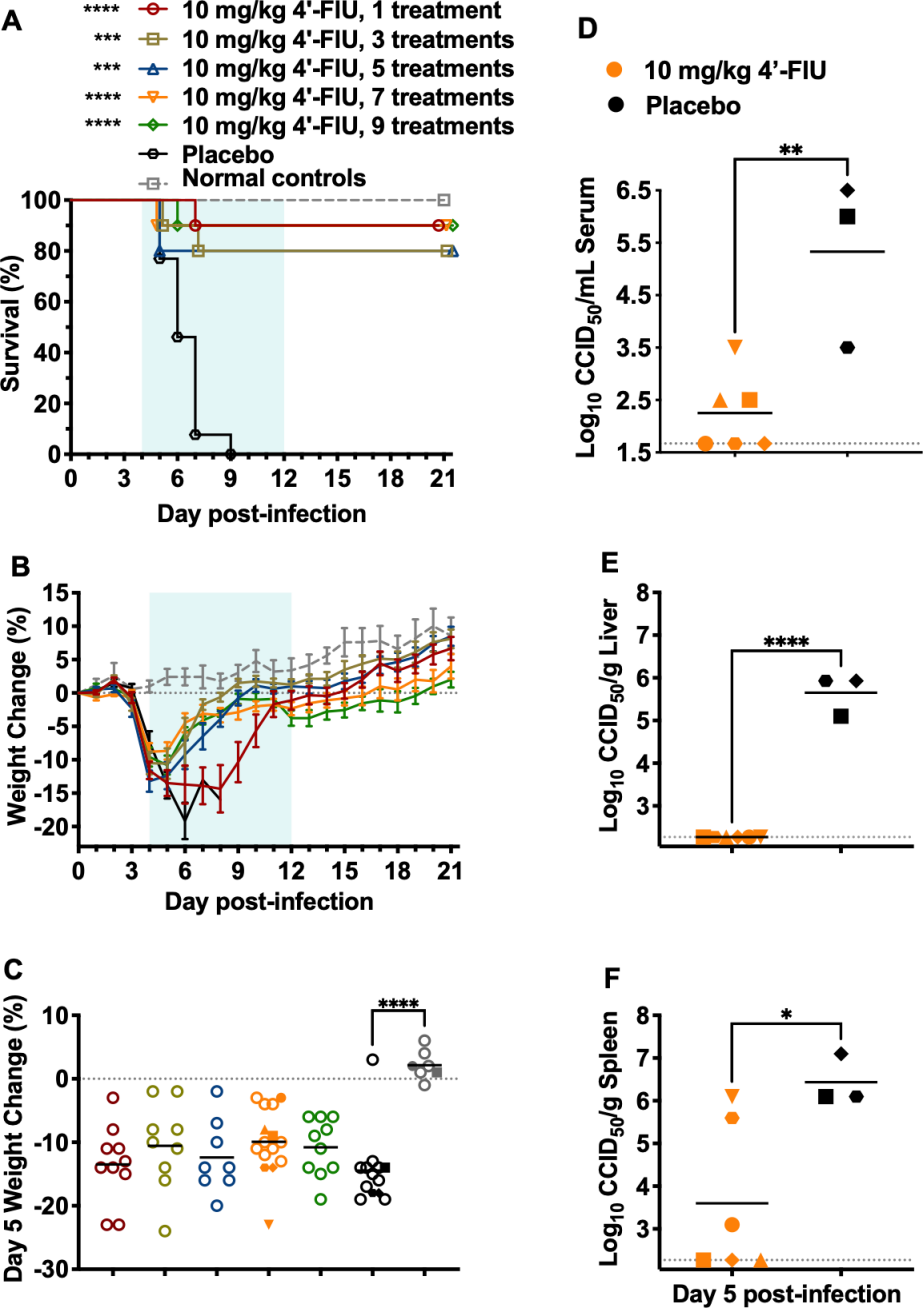
Impact of 4′-FlU dose sparing on DBV-infected *Ifnar*^-/-^ mice when treatment is started 4 days p.i. Animals in each group (*n* = 10/treatment group; *n* = 4 sham-infected normal controls) were treated p.o., QD with 10 mg/kg 4′-FlU starting on day 4 p.i., for the indicated number of days to assess (**A**) survival outcome and (**B**) daily body weights. The weight data are represented as the group mean and standard error of the percent change in weight of surviving animals relative to their starting weights on the day of virus challenge. (**C**) Day 5 weight change from all animals. (**D**) viremia, (**E**) liver, and (**F**) spleen tissue viral titers from pre-selected mice in the 7-day treatment and placebo groups sacrificed on day 5 p.i. Samples could not be obtained from 3 mice in the placebo group. Solid symbols shown in panel C represent the same animals sacrificed for viral titer analyses. Horizontal lines represent the mean for each group. The dotted lines represent the assays’ lower limits of detection. ****P < 0.0001, ***P < 0.001, **P < 0.01, *P < 0.05 compared with animals that received the vehicle placebo.

Therapeutic dosing of 10 mg/kg 4′-FlU provided complete protection against a uniformly lethal DBV challenge when treatment was initiated as late as day 4 p.i. Even when therapy was started 5 days post-DBV infection, statistically significant protection (56% survival) was observed. To determine the minimum number of 10 mg/kg 4′-FlU doses required to protect *Ifnar^-/-^* mice against DBV infection when initiating treatment on day 4 p.i., animals were treated once daily for 1, 3, 5, 7, or 9 days, assessing morbidity and mortality in the observation groups, and viral loads in cohorts taken down on day 5 p.i. As shown in Fig. 5A, 80%–90% of the mice treated with 4′-FlU survived the infection, regardless of the number of treatments administered. Remarkably, even sick mice only receiving a single dose of 4′-FlU on day 4 p.i. were protected (90% survival, *P* < 0.001). In contrast, uniform lethality was seen in mice treated with the vehicle placebo, with most succumbing by day 7 p.i. Notably, all mice experienced clinical illness exemplified by a sharp decline in weight beginning 4 days after DBV challenge (Fig. 5B). However, the animals treated with 4′-FlU began improving within 24 h of treatment, and the groups that received three or more doses rapidly increased in body weight. In contrast, the mice that were only treated once lagged behind in their recovery. All survivors appeared in good health at the end of the 21-day study.

The impact of 4′-FlU treatment on day-5 viremia and tissue viral loads in cohorts of 6 mice per group that received either a single 10 mg/kg dose on day 4 p.i. or the vehicle placebo was also assessed. Significant reductions in viral loads were observed in the liver, spleen, and serum, with virus levels below the detection limit in all liver samples and 50% of the spleen and serum samples (Fig. 5D, E, and F). Notably, the viral loads in the placebo-treated animals are likely underestimated, as 3 of the placebo-treated mice expired before the time of sacrifice due to advanced disease.

### Efficacy of every other day (QOD) 4′-FlU dosing in the OROV infection model

**Fig 6:**
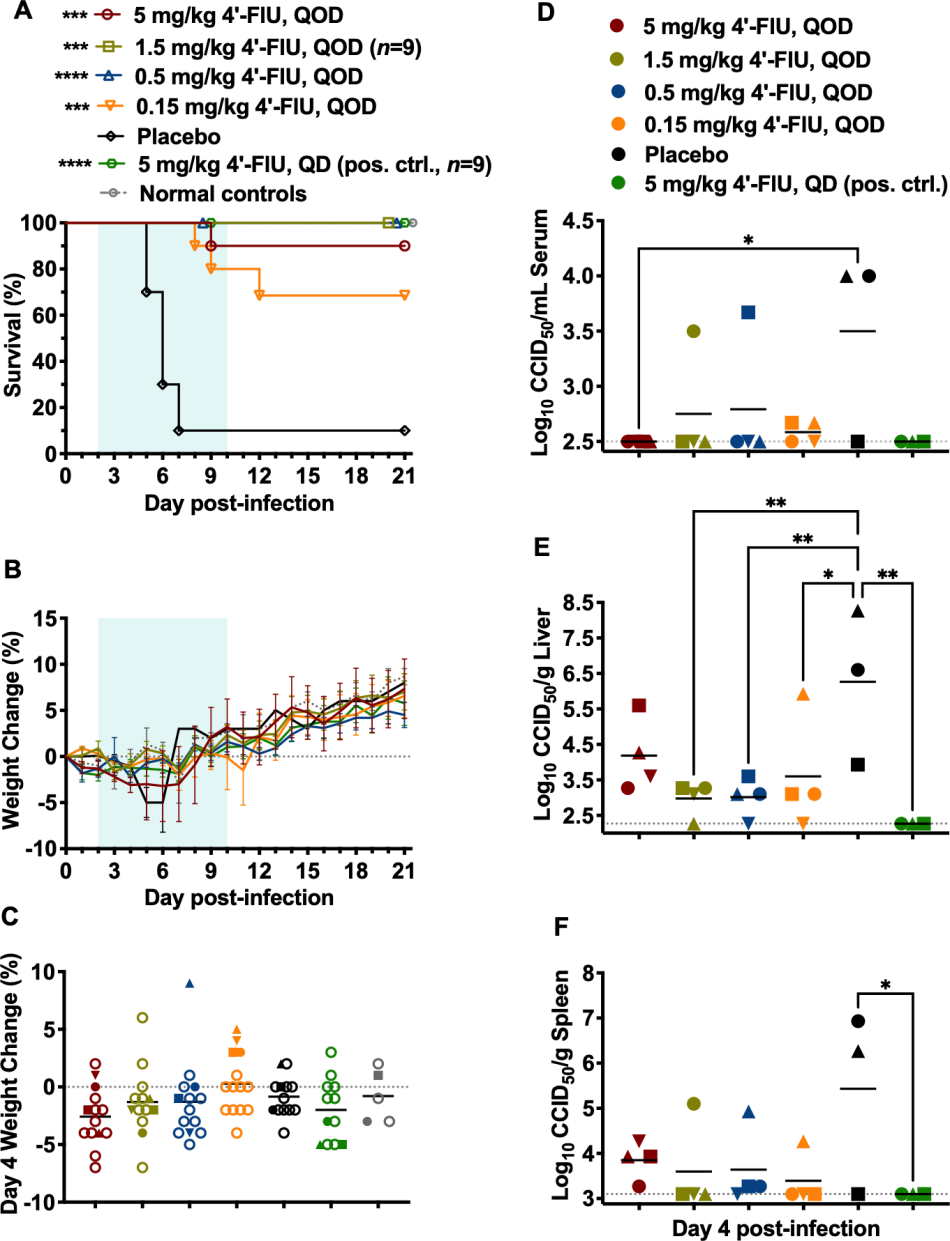
Effect of 4′-FlU treatment administered QOD on *Ifnar* ^-/-^ mice challenged with OROV. Animals in each group (*n* = 10/treatment group unless otherwise indicated; *n* = 4 sham-infected normal controls) were treated p.o., QOD with the indicated dose of 4′-FlU, initiated on day 2 p.i., for 9 days in duration. (**A**) Survival and (**B**) body weights were assessed daily. The weight data are represented as the group mean and standard error of the percent change in weight of surviving animals relative to their starting weights on the day of virus challenge. Sham-infected animals are shown for comparison. (**C**) Day 5 weight change of all animals. Subsets of animals in each group (*n* = 3-4) were predesignated for sacrifice on day 4 p.i. to assess (**D**) serum, (**E**) liver, and (**F**) spleen virus titers. They did not receive the second dose of 4′-FlU. Samples could not be collected from a single mouse in the placebo group, which succumbed before it could be euthanized on day 4. Unique symbols in each treatment group represent values for the same animal across all parameters. Horizontal lines represent the mean for each group. The dotted lines represent the assays’ lower limits of detection. ****P < 0.0001, ***P < 0.001, **P < 0.01, *P < 0.05 compared with animals that received the placebo.

Because OROV (BeAn 19991) has low nM sensitivity to 4′-FlU (Table 1) and a favorable pharmacokinetics (PK) profile in mice (27), we investigated escalating QOD dosing starting treatments 2 days p.i. and continuing for 9 days. As shown in Fig. 6A, all mice treated with 1.5 or 0.5 mg/kg 4′-FlU survived the infection, and even the lowest dose of 0.15 mg/kg offered significant protection (70% survival; ***P < 0.001) compared with the placebo-treated group. In the 5 mg/kg treatment group, a single mouse reached 30% weight loss on day 8 p.i. and, therefore, was humanely euthanized per protocol. Nevertheless, all QOD treatment regimens offered highly significant protection against OROV infection. The body weight data (Fig. 6B and C) were consistent with survival outcomes, and all surviving mice fully recovered with weight gain trajectories that paralleled the sham-infected normal controls.

In cohorts of 3–4 mice per treatment group preselected for sacrifice on day 4 p.i., the impact of a single dose of the 4′-FlU treatments on viremia and tissue viral loads was assessed (Fig. 6D, E, and F). One of the mice in the placebo group succumbed prior to the scheduled euthanasia. Of the remaining three animals, two had viremia near 4 log_10_ median cell culture infectious dose (CCID_50_)/mL serum and greater than 6 log_10_ CCID_50_/g in liver and spleen tissues. Notably, one of the placebo animals had undetectable virus levels, suggesting that disease progression was delayed or the animal controlled the infection. The viral burden was largely kept in check in the 4′-FlU-treated animals. Notably, the mean viral loads for the placebo group are likely an underestimate because the very sick mouse that expired prior to euthanasia could not be included in the analysis.

### 4′-FlU is equally effective at treating more aggressive reassortant OROV infection in *Ifnar^-/-^* mice

**Fig 7:**
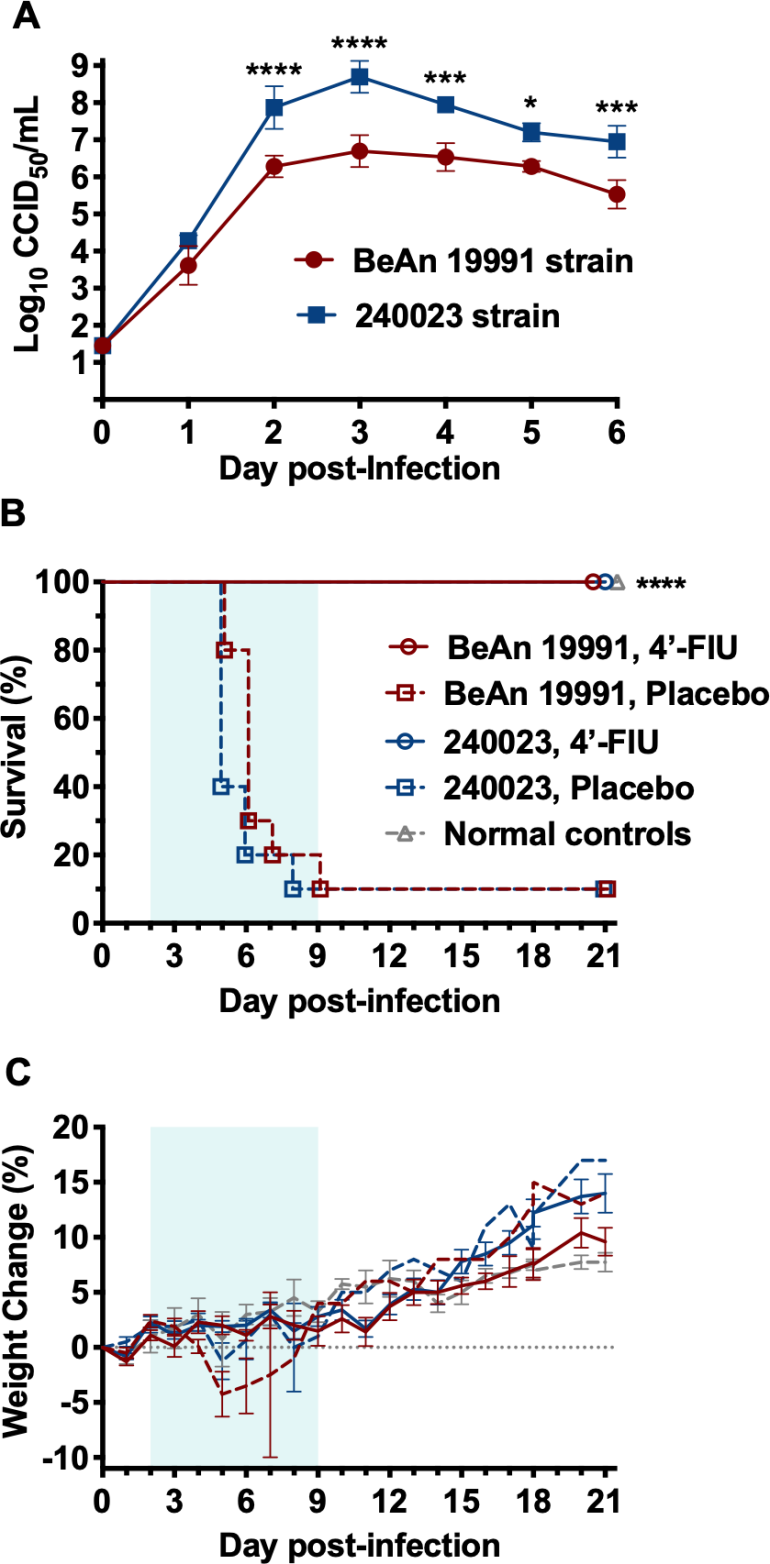
*In vitro* and *in vivo* comparison of the prototypic BeAn 19991 and 240023 reassortant strains of OROV. (**A**) Growth kinetics of both OROV strains in Vero E6 cells infected at an MOI of 0.001. Cell culture supernatants were collected every 24 h for 6 days, and infectious virus concentrations were determined by endpoint titration in Vero E6 cells. ****P < 0.0001, ***P < 0.001, *P < 0.05. (**B**) Survival and (**C**) body weights were assessed daily in *Ifnar^-/-^*mice (*n* = 10/treatment group; *n* = 4 sham-infected normal controls) challenged s.c. with 50 CCID_50_ of the indicated strain of OROV and treated p.o., QD with 3 mg/kg 4′-FlU or the vehicle placebo, initiated on day 2 p.i., for 8 days. The body weight data are represented as the group mean and standard error of the percent change in weight of surviving animals relative to their starting weights on the day of virus challenge. Normal control animals (*n* = 4) are shown for comparison. ****P < 0.0001 compared with respective placebo-treated animals challenged with the same strain.

Because of the ongoing OROV outbreak being driven by a reassortant virus (32, 33), a contemporary epidemic virus strain (240023) from a patient who traveled to Cuba during the current outbreak was obtained, and the efficacy of 4′-FlU was evaluated in parallel with the prototypical BeAn 19991 strain (1960 sloth isolate) used in the studies described above. Although the reassortant 240023 virus grew more rapidly and achieved higher titers in Vero E6 cell cultures (Fig. 7A), *Ifnar^-/-^* mice treated with the vehicle placebo were similarly susceptible to challenge with 50 CCID_50_ of either OROV strain, with 90% of the mice succumbing within 8–9 days p.i. (Fig. 7B). Intervention with 3 mg/kg of 4′-FlU starting 2 days p.i. fully protected the mice against mortality and weight loss caused by both strains, demonstrating comparable sensitivity of the contemporary 240023 reassortant virus with the BeAn 19991 strain (Fig. 7B and C). The potency of 4′-FlU in cell culture was also comparable between the two strains, with the compound having an EC_90_ of 5.6 ± 0.5 nM when tested against the 240023 strain (Table S1 and Fig. S1).

## DISCUSSION

The present study investigated the broad-spectrum prophylactic and therapeutic potential of 4′-FlU to prevent and treat infections by three medically important arboviruses. In Vero cell cultures, the potency of the compound was in the low µM to high nM EC_90_ range for RVFV and DBV, respectively. In contrast, OROV was exquisitely sensitive to the action of 4′-FlU with low nM inhibitory activity. RVFV and DBV are members of the *Phenuiviridae* family within the order of *Hareavirales*, whereas OROV belongs to the *Peribunyaviridae* family under the *Elliovirales* order. The RdRp is the antiviral target of 4′-FlU against several respiratory viral infections (RSV, SARS-CoV-2, IAV, enterovirus) and chikungunya virus (28, 34–36). Presumably, differences in the binding of the 4′-FlU-5′-triphosphate to the phenuiviral and peribunyaviral RdRps targeted by the active form of the drug account for the multiple orders of magnitude difference in sensitivity. Cell culture studies also highlighted the superior potency of 4′-FlU compared with favipiravir, a purine nucleic acid analog approved in Japan to treat SFTS (37, 38) and proven to be effective against RVFV in hamster and rat infection models (39, 40). *In vitro*, 4′-FlU was 16-fold to 29-fold more potent than favipiravir against RVFV and DBV infection in cell culture. In testing against the prototypical (BeAn 19991) and contemporary (240023) strains of OROV, the difference was far more pronounced with 4′-FlU having an EC_90_ in the range of 5.6 to 7.4 nM, which was greater than 2,000-fold more potent than favipiravir (17–18 µM).

Prophylactic efficacy studies are important to demonstrate proof-of-concept and determine the protective dose range of a compound in small-animal models. However, from a practical perspective, individuals typically only seek medical attention after disease onset and, most often, after several days of at-home care. Thus, the infection is well established when the patient presents to the clinic. Although the timing from inoculation to disease presentation in experimental rodent viral infection models is often more accelerated, and it is difficult to extrapolate to the human condition, delaying treatment several days after virus challenge and, in some cases, after the animals are showing signs of illness, speaks to the potential of a compound to be helpful as a therapeutic intervention. Here, we defined the post-exposure and/or therapeutic treatment window for orally dosed 4′-FlU, demonstrating dramatic protection when starting treatments several days after s.c. challenge with RVFV, DBV, and OROV.

In the reported RVFV studies, the peracute nature of the infection in BALB/c mice was underscored by 30%–40% of the animals succumbing to disease within 4 days of virus challenge and 60%–70% mortality by 5 days p.i. Initiating the 10 mg/kg oral 4′-FlU treatments 1 or 2 days after infection protected 90% and 70% of mice against the lethal challenge dose, and those that expired survived far longer, likely dying from late-onset neurologic disease. By comparison, 200 mg/kg/day of favipiravir starting 1 day after RVFV challenge was required to achieve 90% protection. Remarkably, even when delaying 4′-FlU intervention until day 3 p.i., the day prior to when mice begin to succumb to the viral challenge, statistically significant (*P* < 0.05) protection (40% survival) was achieved. Although 4′-FlU does cross the blood-brain barrier, brain tissue exposure is far lower than in the lung, heart, spleen, and liver (27). However, post-exposure treatment can be effective against neuroinvasive avian influenza (27), which is relevant to the treatment of RVFV and possibly other neurotropic infections (9, 10). The results support moving to rat and non-human primate RVFV challenge models to further explore the potential use of 4′-FlU to treat human cases of RVF.

Compared with RVFV in BALB/c mice, DBV infection in *Ifnar^-/-^* mice evolves more slowly, with mice succumbing 6–8 days p.i., following a period of precipitous weight loss that begins 4 days after challenge. Impressively, 10 mg/kg oral 4′-FlU treatments started on or before day 4 of a uniformly lethal DBV infection offered complete protection against mortality. Even more striking was the ability of the compound to rescue 56% of the mice (*P* < 0.05 compared with the placebo treatment) when initiating therapy on day 5 p.i., when one mousein the treatment group had already succumbed to infection. The day 4 and 5 interventions with 4′-FlU truly meet the definition of therapeutic dosing, treating animals with clinically apparent disease. Therapeutic protection with rapid recovery has also been reported in the Heartland virus AG129 mouse infection model and guinea pig models of Lassa and Junín virus challenge (29, 31). In the guinea pig models, the compound’s therapeutic efficacy was punctuated by rapid defervescence within 24 h of the initial treatment. In addition, two therapeutic doses (QOD) of 4′-FlU resulted in significant protection (84% survival) in the Junín virus guinea pig challenge model (29). This finding is consistent with our observation in the DBV challenge model, where 90% protection was achieved in clinically ill mice that received a single drug dose the day before the placebo-treated mice started to succumb to disease. Against a lethal challenge with a pandemic H1N1 influenza reporter virus, the minimal number of doses required to achieve a significant level of protection was one or two doses, resulting in 40% and 80% survival, respectively (27). Taken together, these findings suggest that more abbreviated treatment regimens may be appropriate for certain acute viral diseases.

OROV infection in *Ifnar^-/-^* mice was also responsive to delayed intervention with 4′-FlU. Initiating treatment on day 4 p.i., a time when there was already a death in the placebo group and the day before 30% of mice succumbed to infection, 3 mg/kg 4′-FlU treatment resulted in 80% survival in the face of an otherwise uniformly lethal OROV challenge dose. Beyond the delayed treatment regimens investigated, QOD dosing was explored and found to be highly effective (100% protection) with as little as a 0.5 mg/kg dose. A similar protective effect was reported in the Lassa and Junín virus infection models, with 0.5 mg/kg defined as the lowest dose achieving 100% protection in the latter (29). While these initial studies were conducted using the previously characterized OROV *Ifnar^-/-^* mouse infection model based on viral challenge with the BeAn 19991 strain, we established a new infection model with a contemporary isolate (strain 240023) representative of the new reassortant lineage underlying the current epidemic. Although the 240023 virus grew more vigorously in Vero E6 cell cultures compared with the BeAn 19991 strain, there was little to no difference observed in disease progression and mortality in *Ifnar^-/-^* mice. Importantly, the 240023 OROV was equally sensitive to 4′-FlU treatments in both cell culture and *Ifnar^-/-^* mice.

The continuing OROV disease outbreaks in South American and Caribbean countries underscore the urgency to identify and develop broadly acting antiviral drugs that can be deployed to reduce disease burden and transmission, especially in regions with overlapping presence of viral diseases with similar early symptomology, including dengue, yellow fever, Chikungunya, and/or Zika. In addition to broad-spectrum therapies, rapid diagnostics will be essential to indicate the most effective treatment option for each patient. This is relevant in the context of OROV and Chikungunya virus infections, which respond to 4′-FlU, whereas flaviviruses appear to be refractory to the compound’s antiviral activity. Similar challenges also pertain to SFTS and RVF. While recognizing the limitations of the mouse models evaluated here, the dramatic post-exposure and therapeutic protection observed was remarkable and adds to the expanding list of important viral infections that can be effectively treated experimentally with 4′-FlU, including RSV, seasonal and highly pathogenic influenza viruses, SARS-CoV-2, several arenaviruses (Lassa, Junín, lymphocytic choriomeningitis), and Heartland and Chikungunya viruses (27–31, 41, 42).

## MATERIALS AND METHODS

### Viruses, cells, and animals

The molecular clone of RVFV, strain ZH501 (43), was kindly provided by Dr. Stuart Nichol (CDC, Atlanta, GA), and the virus stock was prepared from a single passage in BSRT7 cells, followed by three passages in Vero E6 cells (African green monkey; ATCC). DBV, strain HB29, and OROV strain BeAn 19991 were obtained from the World Reference Center for Emerging Viruses and Arboviruses (WRCEVA) at the University of Texas Medical Branch. The OROV strain 240023 was obtained from WRCEVA and the Centers for Disease Control and Prevention, Fort Collins. The DBV stock was prepared from two passages in Vero E6 cells. The OROV stocks were derived from a single passage in Vero E6 cells. All virus stocks were prepared from clarified cell culture lysates and diluted in sterile Minimal Essential Medium (MEM) to inoculate the specified challenge doses. All work with RVFV was conducted in enhanced biosafety level 3 (BSL-3+) laboratories by US Federal Select Agent Program-approved personnel at Utah State University (USU). Work with DBV and OROV was performed under conventional BSL-3 containment.

Male and female 18–20 g BALB/c mice were obtained from Charles River Laboratories (Wilmington, MA), acclimated for 7 days prior to the start of RVFV experiments, and fed Harlan Lab Block and tap water *ad libitum*. The DBV and OROV experiments were conducted using male and female 6- to 8-week-old IFN-α/β receptor-knockout (*Ifnar^-/-^*) mice obtained from the breeding colony at USU. They were acclimated for 3–7 days and fed irradiated Harlan Lab Block and autoclaved tap water *ad libitum*. Male and female mice were housed separately.

### *In vitro* evaluation of 4'-FlU by viral yield reduction assay

The sensitivity of RVFV, DBV, and OROVs to 4′-FlU was evaluated by virus yield reduction (VYR). Varying concentrations of 4′-FlU (Emory Institute for Drug Development), ribavirin (ICN Pharmaceuticals, Inc.), or favipiravir (TargetMol) were added to quadruplicate test wells containing 70%–80% confluent Vero 76 (RVFV) or Vero E6 (DBV and OROV) cells just prior to infection at a multiplicity of infection (MOI) of approximately 0.002. Plates were incubated for 4 days, after which virus-infected plates were frozen and thawed, and culture lysate supernatants were collected for endpoint titration of infectious virus. The thawed samples were plated on the respective cell line, and the visual cytopathic effect (CPE) was measured on day 7 p.i. In parallel, cell viability in treated, uninfected cells was measured on day 4 p.i. by neutral red dye uptake assay as previously described (44). VYR data are expressed as the concentration of drug that reduced virus yield by one log_10_ (EC_90_) calculated by regression analysis.

### Evaluation of 4′-FlU as a prophylactic intervention

The 4′-FlU compound was prepared fresh daily in sterile-filtered 10 mM trisodium citrate (Amresco) and sterile injection-grade water at the required concentrations for oral gavage (p.o.) delivery in a 0.1 mL volume. Mice were weighed and sorted to minimize weight and sex differences between treatment groups (*n* = 9–10 per observational group and *n* = 4–5 per group for virological assessments) 1–3 days before 4′-FlU, favipiravir, or placebo dosing. For the prophylactic efficacy studies, QD 4′-FlU treatments were initiated 2 h before the viral infections. Each virus stock was diluted in sterile MEM immediately prior to inoculation by s.c. injection of 0.2 mL.

BALB/c mice challenged with approximately 200 CCID_50_ (as measured by endpoint dilution described in the next section) of RVFV were treated with 15, 5, 1.5, or 0.5 mg/kg of 4′-FlU, or the vehicle placebo, for 7 days. Mice treated with 100 mg/kg favipiravir by i.p. injection twice daily (BID) beginning 2 h before the RVFV challenge and continued for 7 days were included as positive controls.

*Ifnar^-/-^* mice challenged with approximately 30 CCID_50_ of DBV were treated with 15, 5, or 1.5 mg/kg 4′-FlU, or the vehicle placebo, for 9 days. The positive control group received 50 mg/kg favipiravir BID for 9 days by i.p. injection beginning 2 h pre-virus challenge.

Doses of 10, 3, 1, or 0.3 mg/kg 4′-FlU were used to treat *Ifnar^-/-^* mice challenged with approximately 30 CCID_50_ OROV (BeAn 19991) for 7 days. Favipiravir (50 mg/kg; positive control) was administered i.p., BID for 7 days beginning 2 h prior to OROV challenge.

In each of the prophylactic antiviral efficacy studies, 4–5 preselected mice in every group challenged with virus and treated with 4′-FlU were sacrificed on day 4 of the infection to assess the impact of treatment on viremia and tissue (liver and spleen) viral loads.

### Serum, liver, and spleen virus titers

Virus titers were assayed by endpoint dilution using an infectious cell culture assay as previously described (44). Briefly, a specific volume of tissue homogenate or serum was serially diluted and added to triplicate wells of Vero 76 (RVFV) or Vero E6 (DBV, OROV) cell monolayers in 96-well microtiter plates. The viral CPE was determined 5 days (RVFV) or 7 days (DBV and OROV) after plating, and the 50% endpoints were calculated as described (45). In samples presenting with virus outside the detection limits, a value representative of the lower or upper detection limit was assigned for statistical analysis. Notably, for all efficacy studies reported, tissues were collected from mice that were not perfused. Thus, we cannot rule out the contribution of virus from the blood within the homogenized organs.

Back titrations of the virus inoculum preparations used for the challenges were also performed by endpoint dilution and confirmed that the challenge doses were within 10 CCID_50_ of the stated virus doses.

### Post-exposure or therapeutic efficacy of 4′-FlU in RVFV, DBV, and OROV mouse infection models

The 4′-FlU compound was prepared and administered as described for the prophylactic efficacy studies. Similarly, mice were weighed and sorted to minimize weight and sex differences between treatment groups (*n* = 9–10 per observational groups and *n* = 4 per group for virological assessments) 2–3 days before viral challenge. For the therapeutic efficacy studies, QD 4′-FlU treatments were initiated 1-5 days p.i., as specified below and in the figures. Each virus stock was prepared as described for the prophylactic efficacy studies for s.c. inoculation of 0.2 mL.

Groups of BALB/c mice (*n* = 10) were challenged with 30 CCID_50_ of RVFV and treated for 7 days with the vehicle placebo or 10 mg/kg 4′-FlU beginning on days 1, 2, or 3 p.i. An additional treatment group received 15 mg/kg 4′-FlU starting on day 3 p.i. Favipiravir (100 mg/kg) dosed BID by i.p. injection starting on day 1 p.i. served as the positive control.

For the DBV therapeutic efficacy study, *Ifnar^-/-^* mice (*n* = 9/10 per group) were challenged with 30 CCID_50_ of virus and treated for 9 days with 10 mg/kg 4′-FlU beginning on days 1, 2, 3, 4, or 5 p.i. The positive control, 100 mg/kg/day (divided into two daily i.p. doses of 50 mg/kg) favipiravir, was initiated 1 day p.i. and continued for 9 days.

To evaluate post-exposure efficacy of 4′-FlU against challenge with 30 CCID_50_ of OROV (BeAn 19991), infected *Ifnar^-/-^* mice (*n*=10/group) were treated p.o., QD for 7 days with 3 mg/kg 4′-FlU starting on days 1–4 p.i. A group of mice treated with 3 mg/kg 4′-FlU starting 2 h pre-infection was included as the positive control.

In each of the delayed treatment studies, cohorts of 4 mice per group were challenged with virus and treated with 4′-FlU in parallel to the observational groups. These mice were sacrificed on either day 3 (RVFV) or day 4 (DBV, OROV) of the infection to assess the impact of treatment on serum, liver, and spleen viral burden.

### Effect of therapeutic dose sparing on DBV infection outcome

Because of the robust therapeutic efficacy observed with the DBV infection model, a follow-up experiment assessed the minimum number of p.o., QD 10 mg/kg 4′-FlU doses needed to protect *Ifnar^-/-^* mice from a lethal DBV challenge (30 CCID_50_) when initiating treatment on day 4 p.i. In addition, 6 animals from one of the 4′-FlU treatment groups and the placebo group were preselected for sacrifice on day 5 p.i. to determine serum, liver, and spleen viral titers. The remaining animals (*n* = 10/group) were observed for 21 days for morbidity (weight loss) and mortality.

### Assessment of reduced dosing frequency in the BeAn 19991 OROV infection model

Based on the nM *in vitro* potency of 4′-FlU versus OROV and a favorable PK profile (27), escalating doses of the compound were administered p.o., QOD starting on day 2 post-OROV challenge (30 CCID_50_) of *Ifnar^-/-^* mice, with the last of the 5 treatments delivered on day 10. The impact of the different QOD treatments on survival outcome and body weight change (observational groups of 10 mice/cohort) was assessed as before, and 4 preselected animals from each group were for sacrifice on day 4 p.i. to measure viremia and hepatic and splenic tissue viral loads.

### Growth and virulence of contemporary OROV and its sensitivity to 4′-FlU

OROV growth curves comparing the prototypical BeAn 19991 strain and a contemporary 240023 strain were initiated by infections of Vero E6 cells at a MOI of 0.001 in 24-well plates in medium supplemented with 2% FBS and 50 µg/mL gentamicin (Gibco). After 2 h, the infection media was removed, the cells washed, and fresh culture media was added to each well for continued incubation. Culture supernatants were collected from designated wells immediately after adding culture medium (time 0) and every 24 h for 6 days. The supernatant samples were serially diluted in culture medium and added to triplicate wells of Vero E6 cells in 96-well microplates. CPE was assessed microscopically 6 days after infection, and 50% endpoints were calculated. The virus detection limit for the supernatant samples was 1.45 log_10_ CCID_50_/mL. In samples where the virus was undetectable, a value representing the detection limit was assigned for graphical representation.

The virulence of the OROV strains in *Ifnar^-/-^* mice and the treatability of the infections with 4′-FlU were compared side by side. Cohorts (*n* = 10/group) of mice were challenged s.c. with 50 CCID_50_ of one or the other strain of OROV and treated p.o., QD with 3 mg/kg of 4′-FlU or vehicle placebo. The mice were monitored for survival and weight loss for 21 days.

### Statistical analysis

The log-rank test was used for the analysis of Kaplan-Meier survival plots. A one-way analysis of variance (ANOVA) with Dunnett’s multiple comparisons test was used to assess differences in virus titers and single-day body weights. A two-way ANOVA with Šídák’s multiple comparisons test (with a single pooled variance) was used to compare OROV growth curve viral titers. All graphs were generated, and statistical evaluations were performed using Prism 10 (GraphPad Software).

## SUPPLEMENTAL MATERIAL

Supplemental material mbio.01467-25-s0001.docx Figure S1 and Table S1.

ASM does not own the copyrights to Supplemental Material that may be linked to, or accessed through, an article. The authors have granted ASM a non-exclusive, world-wide license to publish the Supplemental Material files. Please contact the corresponding author directly for reuse.
