# Supplementary material for: Effective treatment of advanced Oropouche virus, Rift Valley fever virus, and Dabie bandavirus infections with 4'-fluorouridine
Source: mBio. 2025 Sep 12;16(10):e01467-25. doi: 10.1128/mbio.01467-25 (PMC12505912; doi:10.1128/mbio.01467-25)
Supplement: Supplemental material — Figure S1 and Table S1. [file mbio.01467-25-s0001.docx]

**Supplemental Figure 1. Inhibition of RVFV, DBV, and OROV by 4’-FlU, ribavirin, and favipiravir.** The inhibition curves based 8-concentration VYR assays in Vero 76 cells infected with RVFV or Vero E6 cells infected with DBV, OROV (BeAn 19991 strain) or OROV (240023 strain). The data represent the mean ± standard deviation of 3 separate experiments.

**Supplemental Table 1. *In vitro* inhibition of OROV strain 240023 by 4’-FlU, ribavirin, and favipiravir.**

|  |  | **4’-FlU** | **Ribavirin** | **Favipiravir** |
| --- | --- | --- | --- | --- |
| **OROV**  **(strain 240023)** | **EC_90_ (μM)** | 0.0056 ± 0.0005 | 52 ± 11 | 18 ± 2.3 |
|  | **CC_50_ (μM)** | > 1 | 3500 ± 100 | 2767 ± 208 |
|  | **SI_90_** | > 180 ± 17 | 69 ± 15 | 156 ± 24 |

The inhibitory effect of 4’-FlU, ribavirin, and favipiravir was evaluated in 4-day VYR assays using Vero E6 cells. The data represent the mean ± standard deviation of 3 separate experiments.

EC_90_ = 90% inhibitory concentration. CC_50_ = 50% cell cytotoxic concentration. SI_90_, Selectivity Index = CC_50_/EC_90_.
